# Complex network analysis techniques for the early detection of the outbreak of pandemics transmitted through air traffic

**DOI:** 10.1038/s41598-023-45482-9

**Published:** 2023-10-24

**Authors:** Ángel Fragua, Antonio Jiménez-Martín, Alfonso Mateos

**Affiliations:** Decision Analysis and Statistics Group, Universidad Politécnica de Madrid, 28660 Boadilla del Monte, Spain

**Keywords:** Infectious diseases, Aerospace engineering, Applied mathematics

## Abstract

Air transport has been identified as one of the primary means whereby COVID-19 spread throughout Europe during the early stages of the pandemic. In this paper we analyse two categories of methods – dynamic network markers (*DNMs*) and network analysis-based methods – as potential early warning signals for detecting and anticipating COVID-19 outbreaks in Europe on the basis of accuracy regarding the daily confirmed cases. The analysis was carried out from 15 February 2020, around two weeks before the first COVID-19 cases appeared in Europe, and 1 May 2020, approximately two weeks after all the air traffic in Europe had been shut down. Daily European COVID-19 information sourced from the World Health Organization was used, whereas air traffic data from *Flightradar24* has been incorporated into the analyses by means of four alternative adjacency matrices. Some *DNMs* have been discarded since they output multiple time series, which makes it very difficult to interpret their results. The only *DNM* outputting a single time series does not emulate the COVID-19 trend: it does not detect all the main peaks, which means that peak heights do not match up with the increase in the number of infected people. However, many combinations of network analysis based methods and adjacency matrices output good results (with high accuracy and 20-day advance forecasts), with only minor differences from one to another. The *number of edges* and the *network density* methods are slightly better when dynamic flight frequency information is used.

## Introduction

The Coronavirus pandemic (COVID-19) has brought about countless changes worldwide since its onset in early 2020. The World Health Organization (WHO) declared COVID-19 an international public health emergency on 30 January 2020 and a global pandemic on 11 March 2020. It was at this point that most countries began to establish more stringent control measures both at the international level (such as air traffic restrictions and border controls) and at the local level (cessation of public transport, closure of public institutions, quarantine of the population, etc.).

In global pandemics, such as COVID-19, two phases in the spread of the virus can be distinguished. At the beginning, countries face the challenge of preventing the virus from entering their country. This international transmission is one of the most important phases, as controlling the arrival of the virus in the country can mitigate its spread and thus reduce the multiple (social, health, economic, etc.) impacts on the population. If not properly managed during international transmission, it is likely that within the same country there will be so many carriers of the virus that it will be impossible to determine the origin of the infections and therefore the chain of infection will be broken. It is at this point that the phase known as community transmission begins.

Community transmission^1^ is a critical indicator for a country, as there is no control over the spread of the disease within its borders. Therefore, severe measures such as social distancing, the use of masks and even total quarantine of the country are the only option. The most restrictive interventions, such as quarantines, are highly effective in blocking and mitigating the spread of the virus, but at the same time they delay health interventions such as vaccination and cause great social, political and economic instability within the country. Such measures should be avoided by better management of international transmission, and attempts to prevent uncontrolled transmission between all the European Union countries.

One possible control measure is the analysis of early warning signals for the detection of the global spread of the virus. The main modes of spread of the virus may include air, sea and land transport in all its forms. The impact of each type of transport in different regions, countries and continents will differ. In Europe, air transport has been identified as one of the primary means of the international spread of COVID-19. This fast and inexpensive mode of transport can be seen as a double-edged sword. This is because, while it allows connections between remote places, facilitating further cultural and economic development, it also facilitates the export and import of new diseases between distant places that would otherwise never have occurred.

Due to the COVID-19 pandemic, there have been a plethora of papers related to all sorts of different disciplines, such as health sciences, social sciences, computer simulations, economics, Social Network Analysis (SNA), among many others. The sole purpose of several papers is to organize and categorize all this information in reviews^2,3^, with the spread of the virus through different means of transport, especially air traffic, being one of the most studied topics.

Most publications on the calculation of the risk of virus spread through air traffic focus their efforts on applying the susceptible-infectious-recovered (SIR)^4^ (originally proposed by Billio et al.^5^) and its many variants^6^, e.g. susceptible-exposed-infectious-recovered (SEIR)^7^, susceptible-exposed-infectious-recovered-susceptible (SEIRS)^8^, susceptible-infectious-susceptible (SIS)^9^ or airport-based susceptible-infected-recovered-susceptible (ASIRS)^10^, among others. The drawback of such models is their focus on quantifying the risk of virus transmission in closed populations. However, there are other variants of analysis that focus on mathematical and statistical models, based on the creation and analysis of complex static or dynamic networks. Instead of focusing on the spread of the virus within a closed population or region, they attempt to understand the effect of the spread through a global approach to the pandemic, and warning signs can be detected prior to the spread of the outbreak.

Some of the most prominent papers in the literature on complex network analysis focused on COVID-19 spread through air transport are the following:

Tiwari et al.^11^ analyse the risk of COVID-19 outbreak spread in the United States (US) through the analysis of complex networks and air traffic, comparing the time series of network density, number of new daily confirmed COVID-19 cases and frequency of flights between the five states most affected by the pandemic. The dynamic network used for the density is an undirected, unweighted network, where each node represents a US state, and the links between each pair of states represent the square root correlation of their changes in the number of confirmed COVID-19 cases over the past 14 days. If the correlation between two states exceeds the 0.5 threshold, the connectivity between them is established. Its ultimate goal is to find patterns and trends in COVID-19 pandemic connectivity. The design and generation of these networks provides a way to visualize the global spread of pandemics and their risk of expansion^12^. The analysis of these networks, in particular the degree of connectivity between nodes according to the number of confirmed cases of COVID-19, provides a powerful tool for assessing the risk of global spread and even its early detection.

So et al.^13^ expands on Tiwari et al.^11^ proposing new measures of global connectivity in addition to network density for analysing global pandemic risk using graph analysis, such as the number of links, the *clustering coefficient*, or the *assortativity coefficient*. Moreover, they propose two new measures for measuring the risk of a pandemic spreading, the *Preparedness Risk Score* and the *Severity Risk Score*. Following the same methodology, Chu et al.^14^ assess why it would be appropriate to revise the air restrictions using network density for the analysis of the overall risk of pandemic spread. The work concluded that a new wave of contagion was quite plausible. Chu et al.^15^ study the impact of air restrictions in Latin America using the connectivity network of the correlation between the number of infected people. The study shows that Latin American air hubs match up with the first countries with positive COVID-19 cases and that applying restrictions on these airports would lead to a decrease in the overall connectivity of the network.

Chu et al.^14^ explain in detail the whole workflow for the creation of a spatio-temporal air traffic database. They aim at building a dynamic network where each instant is represented by a graph for measuring the connectivity between nodes (whether airports, countries, regions) based on the frequency of flights between nodes. Sun et al.^16^ perform a similar analysis to Chu et al.^14^ on the spatio-temporal evolutionary dynamics of COVID-19 in the global air transport network. For the construction of the dynamic network of air traffic connectivity, they propose two alternatives: one where the nodes are airports and another where the nodes are countries, i.e. each country groups all the connections of its airports. The links simply indicate the existence of direct flights between two nodes. Among the properties of the graphs taken into account for the study of evolution are the average degree centrality, the average betweenness centrality and the degree of assortativity. Based on these same ideas, Sun et al.^12^ investigate the degree of synchronization between the number of confirmed cases and the air containment measures applied in each country. The result shows that most countries closed their borders late, making the impact of the measures less efficient.

An alternative methodology, which studies the temporal evolution of a network by analysing the graph corresponding to each time instant, is proposed by Zhu et al.^17^. A single graph is generated, where the nodes represent countries, and the links represent the correlation to the monthly growth rate of infected people. Since there is only one graph, they can afford to study centrality measures and other global network measures, such as the clustering coefficient, the average degree of connectivity between the nodes, the average distance between the nodes and the diameter of the network. Finally, they study the impact of certain social and economic factors on COVID-19 connectivity by applying quantitative regression methods.

Barros De Souza et al.^18^ explains a parameter-free mechanism based on the Forman-Ricci curvature, which, like the rest of the dynamic networks calculated based on the correlation coefficient of confirmed COVID-19 cases between each pair of nodes, studies a measure of the connections or links between the nodes. The reason why the Forman-Ricci curvature is used instead of the Ollivier-Ricci curvature in this paper is that it has been shown that both have analogous properties^19^, but the Forman-Ricci curvature stands out for its reduction in the computational time for large-scale problems and real problems with highly complex graphs. In this work, the correlation coefficient corresponding to all existing links is calculated, but they are added in descending order until the state of the network known as *giant component*^20^ is reached. This state implies that all nodes are connected to each other, and, therefore, there is at least one path between each pair of nodes forming a single cluster.

Finally, Dynamical Network Markers (*DNM*)^21^ are based on a set of general criteria based on dynamic networks that allow the detection of early warning signals, i.e. the detection of impending network bifurcation before the tipping point or entry into the critical state occurs. The *Minimum-Spanning-Tree-based Dynamical Network Marker* (*MST-DNM*) is the only method that has been applied to the COVID-19 expansion problem by Dong et al.^22^. In this work, this same marker is applied to successfully detect early warning signs of COVID-19 among different regions of Italy based on the number of confirmed cases they present.

The aim of this paper is to analyse two categories of methods in the literature – dynamic network markers (*DNMs*) and network analysis-based methods – as potential early warning signals for detecting and anticipating COVID-19 outbreaks at an early stage of the pandemic in Europe on the basis of accuracy regarding the confirmed daily cases. The analysis was carried out from 15 February 2020, around two weeks before the first COVID-19 cases appeared in Europe, and 1 May 2020, approximately two weeks after all the air traffic in Europe had been shut down.

Daily European COVID-19 information from the World Health Organization was transformed into a complex networks, one for each instant in time, and a complex network analysis is applied to the generated data set. The impact and relationship of air traffic in Europe on the spread of the virus is also analyzed, accounting for both the frequency of flights between pairs of European countries and the number of flights. Air traffic data from *Flightradar24* was incorporated into the analyses by means of four alternative adjacency matrices.

This early detection of COVID-19 spread will serve to alert the competent authorities or experts in the field, so that they can take appropriate measures to prevent the future uncontrolled spread of pandemics worldwide. An early warning alert of the spread of pandemics can give specialists more time to make decisions and take appropriate actions.

To achieve the overall objective, the following sub-objectives have been established:

- Transformation of the temporal signals of COVID-19 progress, together with country and aerial data, into early warning signals. To do this, graphs must first be generated for each time instant, so that their properties can then be extracted as the final signals.
- Detection of different hyperparameters when generating the graphs and study of their impact on the warning signal.
- Implementation in Python, with the incorporation of result visualization tools.
- Analyse the accuracy and anticipation of the early warning signals with respect to the COVID-19 confirmed case data through a linear regression, and a time series distance measure, the dynamic time warping.

This paper is structured as follows. In the next section, the early warning signal methods proposed in the literature and to be analysed are briefly described, categorized into *network statistics* and *dynamic network makers*. Section "Numerical analysis" is focused on the numerical analysis of early warning signal methods. First, the data used for analysis is described. Then, we explain the incorporation of flight data to the dynamic networks, and, finally, the results of the analyses are reported and discussed, in terms of both accuracy and anticipation. Finally, the conclusions are drawn.

## Early warning signals methods

The generation of early warning signals in the literature is based on the construction of dynamic pandemic networks, which are composed of a specific network for each day of study. Each network is a graphical representation of the COVID-19 evolution, where nodes represent the countries of study, and the edges/links connecting two countries represent the correlation of the changes of the confirmed cases within each country..

The methods for determining early warning signals fall into two main groups. The first group, *network statistics*, extracts well-defined global properties from the dynamic networks such as network density, global clustering coefficient... or other new measurements that have appeared due to this pandemic. The second group, *Dynamical Network Markers* (*DNM*), takes advantage of the similarity between the dynamical process of a pandemic, and the three common stages of disease progression^21^: (i) normal stage, where the disease is under control but in an incubation period; (ii) pre-outbreak stage, where the disease is unstable in-between the normal stage and the tipping point, where there is still a chance of reversibility; (iii) outbreak stage, which is the stage beyond the tipping point where the disease is uncontrollable and the outbreak irreversible uncontrollable and irreversible period is reached once the tipping point is crossed. DNMs can detect the transition from the normal to the pre-outbreak stages in order to apply the proper measures to try to prevent the outbreak stage.

The main difference between both groups is that the early warning signals obtained through network statistics tend to emulate the trend in confirmed COVID-19 a number of days in advance, whereas *DNM* markers do not follow this pattern and stand out by frequently fluctuating or having high peaks previous to the main infection points, which can be detected with the fold-change measure also known as *X*-fold increase, because it checks if there is an increase or decrease of proportion *X* between the value of time *t* and the $t+1$.

### Network statistics

Methods in the literature classified as network statistics share the common trait of generating and analysing dynamic networks by means of different network global properties or well-defined formulas.

Let $X_{i,t}$ be the number of confirmed COVID-19 cases of the country *i* in day *t*. To study the COVID-19 evolution between two countries, we should focus on the daily changes, which can be calculated as $Y_{i,t} = X_{i,t} - X_{i,t-1}$. Another alternative is what is commonly known as the daily changes in the square-root, $Y_{i,t} = \sqrt{X_{i,t}} - \sqrt{X_{i,t-1}}$, which statistically^23^ should make counts more stable. We checked the square-root option but it was discarded since it output the worst results.

For the generation of the pandemic network of day *t*, we need to determine the value of each edge connecting the country *i* and the country *j*. This will be calculated thanks to the correlation $\rho _{ij, t}$ using *k* observations, starting at the day *t* and going backwards (e.g. using the Pearson correlation $\rho _{ij, t} = PearsonCorrelation(\{Y_{i, t} ... Y_{i, t - k}\}, \{Y_{j, t} ... Y_{j, t - k}\})$). This means that for each network of day *t*, we take a sliding window with the data of the day *t* and the $k - 1$ previous days. Applying the correlation for every pair of countries (*i*, *j*) at each instant *t* of interest, we obtain the directed network defined by the matrix $P_{n\_countries \times n\_countries, t}$. This directed network can be easily transformed into an undirected network matrix $G_{n\_countries \times n\_countries, t}$ thanks to a threshold *r*, which usually has a value of around 0.5, where

1 $$ G_{{ij,t}}  = \left\{ {\begin{array}{*{20}l}    {1,} \hfill & {{\text{if}}\quad P_{{ij,t}}  \equiv \rho _{{ij,t}}  > r,} \hfill  \\    {0,} \hfill & {{\text{otherwise}}{\text{.}}} \hfill  \\   \end{array} } \right. $$

To measure the network connectedness, or to summarize its properties, it is mandatory to extract a unique value for each day or network. For this purpose, we define each resultant graph or network at day *t* as $G_t = (V_t, E_t)$, where $V_t = \{v_1, v_2, ..., v_n\}$ represents the group of $n = |V_t|$ vertices, and $E_t = \{e_1, e_2, ..., e_m\}$ denotes the set of existing edges or links with a total size of $M= |E_t|$. Knowing that the edges are unique, which means that there can only be one edge between two vertices, they can also be represented as $e_{ij} = (v_i, v_j)$, which indicates that $e_{ij}$ connects the countries *i* and *j*.

The summarization of the dynamic networks consists of transforming the dynamic networks into a time series with a unique value for each network day. A brief description of the measures that we have considered follows:

*Number of edges.* One of the easiest means of summarizing a network at day *t* is simply counting the number of edges (*m*):

2 $$\begin{aligned} m = |E_t|. \end{aligned}$$

*Network density.* The network density, $D_t$, measures the proportion between the number of existing connections and the maximum number of possible connections that the graph of day *t* could have. If we consider that the graph we are studying has a complete adjacency, which means that every node can be connected to every other node except itself, the maximum possible number of connections will be $(|V_t| (|V_t| - 1)) / 2$ leading to

3 $$\begin{aligned} D_t = \frac{2 |E_t|}{|V_t| (|V_t| - 1)}. \end{aligned}$$

*Clustering coefficient.* The clustering coefficient^24^ of a graph determines the degree to which its vertices tend to cluster together. This measure has subtle changes in its formula depending on the type of graph. In this paper, all graphs are undirected, whose only difference with a directed graph is that they can only have half of its edges. In compensation, the formula has been multiplied by two. This measure was initially intended for nodes, but it can be expanded into a graph global property if the mean is applied for every node that it contains. The clustering coefficient of a node measures the degree of all its neighbors (all accessible nodes by only one edge) to become a *clique* (complete graph).

Let $N_{i, t} = \{v_j: \exists e_{ij} \vee e_{ij} \in E_t\}$ be the group of neighbors of the vertex $v_i$ at day *t*, which is equal to all vertices $v_j$ that are directly connected to $v_i$ by only one edge. The number of neighbors can be represented as $|N_{i, t}|$, and it is normally called degree of a node. The clustering coefficient $c_{i, t}$ of the node $v_i$ at day *t*, determines the proportion between the number of edges of the neighbors of $v_i$ and the maximum number of edges that could potentially exist:

4 $$\begin{aligned} c_{i,t} = \frac{2|e_{jk}: v_j, v_k \in N_{i, t}|}{|N_{i, t}|(|N_{i, t}| - 1)}. \end{aligned}$$

The value range of the clustering coefficient of a node $c_{i, t}$ is [0, 1]. 0 implies that $v_i$ cannot conform a clique at all, and 1 means that it forms a single clique. Once it is understood how the clustering coefficient of a node $c_i$ is calculated, we can simply apply the same formula for each node and summarize it with the mean:

5 $$\begin{aligned} C_t = \frac{1}{|V_t|} \sum _{i=1}^{|V_t|}c_{i, t}. \end{aligned}$$

*Assortativity coefficient.* The assortativity coefficient measures the similarity between all the nodes of a graph, in other words, if the nodes attached to others are similar somehow. For this specific measure, there are plenty of variations, but the one selected for the numerical analysis was originally proposed by Newman^25^. The objective is to measure the correlation between each pair of nodes, and this is obtained using the degree or number of neighbors $|N_{i, t}|$ of each node *i* at time *t*. The assortativity coefficient can be formulated as:

6 $$\begin{aligned} AS_{t} = \frac{\sum \nolimits _{e_{ij}\in E_t} (|N_{i, t}| - \overline{|N_{i, t}|})(|N_{j, t}| - \overline{|N_{j, t}|})}{\sqrt{\sum \nolimits _{e_{ij}\in E_t} (|N_{i, t}| - \overline{|N_{i, t}|})^2}\sqrt{\sum \nolimits _{e_{ij}\in E_t} (|N_{j, t}| - \overline{|N_{j, t}|})^2}}, \end{aligned}$$

where $\overline{|N_{i, t}|}$ refers to the mean of the degree of all input nodes, whereas $\overline{|N_{j, t}|}$ is the mean of the degree of all output nodes. The value range of the assortativity coefficient $AS_{t}$ is $[-1,1]$. Value 1 is associated with the maximum degree of assortativity, whereas -1 represents the minimum.

*Forman-Ricci curvature.* The Forman-Ricci curvature has previously been applied to the economic sector^26^ as an indicator of the market fragility using temporal series. This problem is very similar to the one we are dealing with, being introduced into the epidemiology world^18^ as a means to measure the fragility and systematic risk of epidemic networks. The Forman- Ricci curvature is equivalent to the Olivier-Ricci curvature, since both share analogous properties^19^. The main difference between them, and the reason we prefer one over the other for the numerical analysis, is that the Forman-Ricci curvature is a more efficient (faster) approach from a computational point of view.

The Forman-Ricci curvature $f_{e, t}$ at day *t* is defined for each edge $e \in E_t$ of a graph. We can define it as follows:

7 $$\begin{aligned} f_{e, t} = |cliques\_containing\_e| + 2 - |parallel\_edges\_to\_e|, \end{aligned}$$

where $|cliques\_containing\_e|$ are the number of cliques (simultaneous connections between three nodes, also known as triangles) that contain *e*, and $|parallel\_edges\_to\_e|$ are the number of edges that are sharing either a node or a clique with *e*.

The Forman-Ricci curvature $f_{e, t}$ is intended for edges, but it can easily be extended to the whole graph, making it a global property by applying the mean to all edges at time *t*:

8 $$\begin{aligned} {FormanRicci}_t = \frac{1}{|E_t|} \sum _{e=1}^{|E_t|}f_{e, t}. \end{aligned}$$

*Preparedness Risk Score (PRS).* The main fact about the *PRS*^13^ measure is that it is not a consolidated global graph property described above. Instead, it is a new procedure to determine the potential risk of transmission between countries in a pandemic by using dynamic networks. This new measure is characterized by adding information on the susceptible population, assuming that people that have already been infected with the COVID-19 are highly unlikely to be infected again. To process this measure, we need the matrix $V_{n\_countries, t}$ of confirmed COVID-19 cases at day *t*, and the array of population $P_{n\_countries \times 1}$, where $P_i$ indicates the population of the country *i*. With these matrices, we can obtain the susceptible population by $S_{n\_countries, t} = P_{n\_countries \times 1} - V_{n\_countries, t}$ for day *t*.

We can define the *PRS* as follows:

9 $$\begin{aligned} PRS_t = S_{n\_countries \times 1, t}^{T} G_{n\_countries \times n\_countries, t} S_{n\_countries \times 1, t} = \sum _{i}\sum _{j} S_{i, t} G_{ij, t} S_{j, t}, \end{aligned}$$

where $G_{ij, t}$ was defined in Equation (1).

The first representation of the above equation is a matrix multiplication with size $(1 \times n\_countries)(n\_countries \times n\_countries)(n\_countries \times 1)$ resulting in a single value, whereas the second one is its transformation into a flat operation.

### Dynamic network marker (*DNM*)

There is solid evidence to suggest that during the evolution of complex diseases, the deterioration is not normally progressive. Instead, they are abrupt, causing a transition from a controlled into an uncontrolled state, known as the *tipping point*. Taking advantage of this, the Dynamic Network Markers (*DNM*) can be used to detect early-warning signals for pandemics. Note that these methods are model-free and do not require any training; they work well even with few samples.

These indices are derived from the Dynamic Network Biomarker (*DNB*), previously used to detect the tipping point in some diseases using information in the form of microarrays of genes or proteins. Some examples of the application of *DNB* are: the early detection of Influenza A^27^ studying the evolution of the hemagglutinin protein as an amino acid sequence; the early detection of some diseases like acute lung injury, acute corneal trauma and breast cancer thanks to an approach based on an unsupervised hidden Markov model^28^.

Even though the *DNB* were initially designed as biological markers, and their main focus was the study of complex diseases through the evolution of genes, proteins and other molecules; they have been extended to other disciplines, such as dynamic network markers. In this case, the *DNM* will use the daily number of confirmed cases of a disease to study its expansion between some regions.

As previously mentioned, both the *DNB* and the *DNM* divide the problem (disease or pandemic) into three stages:

- Normal stage: This is the stage where the situation is under control and probably in an incubation period.
- Pre-disease or pre-outbreak stage: This is the stage in-between the normal stage and the tipping point, where the situation can still be reversed to the normal stage if treated properly.
- Disease or outbreak stage: This is the stage beyond the tipping point, where the situation cannot be reverted to the normal stage.

It is obvious when the outbreak stage has started, by simply looking the drastic increase in the number of patients. However, finding the pre-outbreak stage is a challenging task, because there are no significant changes with the normal stage. Theoretically, both *DNM* and *DNB* state that when a dynamic system is close to the tipping point, a group of variables exist known as *DNM* features, which satisfy:

- The correlation $\rho _{in}$ (Pearson correlation) among the pairs of members in the *DNM* group rapidly increases.
- The correlation $\rho _{out}$ between any member of the *DNM* group and any other member of the non-DNM group rapidly decreases.
- The standard deviation, $sd_{in}$, for each member of the *DNM* group drastically increases.

Some specializations of the *DNM* used in the early detection of different pandemics are the following:

- *Minimum Spanning Tree - Dynamic Network Marker (MST-DNM)*. The main idea of this marker is to summarize each network conforming the dynamic network by adding the weight of each edge forming the minimum spanning tree. This is the only marker applied to COVID-19 and exclusively in the region of Italy^22^. It was also applied to forecast the Influenza outbreak in Japan^29^.
- *Shortest Path - Dynamic Network Marker (SP-DNM)*. Another variant of this type of markers, is the one using the average of the shortest path between the main nodes of the graph. In case all nodes have the same priority, it is recommended to use the 4 paths conformed by the main cardinal points: the northernmost with the southernmost node, the easternmost with the westernmost node, the northeasternmost with the southwesternmost node, and the northwesternmost with the southeasternmost node. It has been applied to detect the outbreak of Influenza in Japan^30^.
- *Landscape - Dynamic Network Marker (L-DNM)*. This method outputs a value for each node rather than just one for the whole network representing each instant of time, which is what makes it different from MST-DNM and SP-DNM. This leads to a group of time series instead of a single one resulting in a 3D landscape representation. This marker has already been applied to the early detection of Influenza in Japan^31^, and also to detect the pre-outbreak signals of hand, foot and mouth diseases in Japan^32^.

## Numerical analysis

As described in Section "Early warning signals methods", different models from the literature have been used to construct a dynamic network and subsequently generate and analyze the early warning signals. These networks contain a total of 46 nodes representing the member countries of the Council of Europe^33^ (Albania, Andorra, Armenia, Austria, Azerbaijan, Belgium, Bosnia and Herzegovina, Bulgaria, Croatia, Cyprus, Czech Republic, Denmark, Estonia, Finland, France, Georgia, Germany, Greece, Hungary, Iceland, Ireland, Italy, Latvia, Liechtenstein, Lithuania, Luxembourg, Malta, Monaco, Montenegro, Netherlands, North Macedonia, Norway, Poland, Portugal, Republic of Moldova, Romania, San Marino, Serbia, Slovak Republic, Slovenia, Spain, Sweden, Switzerland, Turkey, Ukraine and United Kingdom).

As the main focus is on early detection (and not a long-term prediction), we will take a time interval between the 15 February 2020, around two weeks before the first COVID-19 cases appeared in Europe, and 1 May 2020, approximately two weeks after all the air traffic in Europe had been shut down. Flight information from *Flightradar24* was used, whereas the two major sources of world daily COVID-19 information are the World Health Organization (*WHO*)^34^ and the Johns Hopkins University (*JHU*)^35^. Even though the code allows the use of any source of information, we opted to use the *WHO* dataset because, after visualization of the two, we detected some “outliers” or strange transitions from one day to another in the *JHU* database (e.g. in the *JHU* database between the 11st and the 13rd of April 2020, the number of confirmed COVID cases fluctuated from 30,000 to 72,000 and back again to 26,000 from 11 to 13 April 2020 in the JHU database, while all the fluctuations are much smoother in the WHO database). Besides, all the fluctuations are much smoother in the WHO database, as proposed by Zhan et al.^36^, could improve the analysis carried out.

The early warning signals methods analyzed were explained in Section "Early warning signals methods": *network statistics*, including number of edges, network density, clustering coefficient, assortativity coefficient, Forman-Ricci curvature, and *PRS*; and *dynamic network markers*, including *MST-DNM*, *SP-DNM* and *L-DNM*.

Regarding the considered methods, we fixed two hyperparameters. The first one is the size of the sliding window *k*, in other words, how many days are being taking into account for the generation of the network of each instant. This was set to $k=14$ days, since this is the most extended value in the literature, corresponding to the quarantine time due to the virus incubation period. In addition, Pearson correlation $\rho _{ij, t}$ has been used in the analyzed methods.

### Incorporation of air traffic data to the dynamic networks

In the literature, all analyses take for granted a complete adjacency between all nodes (except the paper of Dong et al.^22^, where nodes only connect with other nodes if they are adjacent by geographical means), which means that every node or country could be reached by any other. This type of adjacency might be too pessimistic, because there are many countries with practically zero exchange of people, making the transmission of the virus impossible.

To improve the generation of early warning signals, we have developed three novel types of adjacency different from the global adjacency, providing a more realistic representation of Europe’s graph. To do this, the adjacency of the dynamic graph will be tuned using the flight data, since this is the primary form of conveyance in Europe.

Specifically, we provide a graph for each day *t* of study $N_{n\_countries \times n\_countries, t} = (V_t, E_t)$ is available, which is either equivalent to $P_{n\_countries \times n\_countries,t}$ in the case of a network statistic method or is generated by a *DNM* method. To apply a specific adjacency matrix $A_{n\_countries \times n\_countries, t}$ on day *t*, we simply need to apply the Hadamard matrix product (also known as Schur^37^ product or element-wise product) $J_t = N_t o A_t$. Note that $A_{n\_countries \times n\_countries, t}$ is a binary matrix and that $J_t$ will contain the values originally in $N_t$ for elements with value 1 in $A_t$, whereas a value 0 will appear for elements whose value in $A_t$ is 0, i.e. $A_t$ plays the role of a filtering matrix.

We will consider four cases depending on the type of adjacency matrix used:

- *Static complete adjacency*: Static adjacency denotes that all the adjacency matrices $$A_t$$ regardless of the instant *t* of study will be identical. For the specific case of complete adjacency, all possible connections between any pair of nodes are taken for granted, which means that $$A_{ij, t} = 1 $$ with $$ i \ne j$$ ($$A_{ii, t} = 0$$). Thus, $$J_t$$ is the same as $$N_t$$ but including values 0 in the main diagonal.
- *Static flight adjacency*: This is another type of static adjacency. However, in this case, each possible connection between two nodes will be $A_{ij, t} = 1$ only if the number of flights at the time of study exceed a threshold, otherwise $A_{ij, t} = 0$. Besides, $A_{ii, t} = 0$ for every selfconnection. This threshold is used to control when an arc is considered, for example, when there is one or more flights, when there are 50 or more flights, when there are more than the average number of flights, etc. The default value is the average number of flights. Now, only the arcs in the European air traffic graph where the number of flights exceeds a certain threshold are considered in $J_t$.
- *Static flight frequency adjacency*: This static adjacency uses the flight frequency for every adjacency matrix at time *t* during the time of study. Unlike the above, the matrix is divided by the total number of flights instead of establishing threshold to determine whether or not the connection is rejected. This type of adjacency with float values representing the node’s adjacency instead of $0's$ and $1's$ reduces the threshold range [0.0001, 0.005] needed to transform the weighted graph $A_{t}$ into the unweighted and final graph. Now, flight frequency is defined by the weights of the arcs in $J_t$ instead of by the number of flights.
- *Dynamic flight frequency adjacency*: Unlike all the other types of adjacencies, the dynamic adjacency uses only the flight data of the window corresponding to the instant *t* of study, with a completely different adjacency matrix $A_{t}$ for each instance. As the name suggests, each $A_{t}$ corresponds to the static flight frequency adjacency of the window of the instant *t*. A priori, thanks to the introduction of the dynamic adjacency generation and the use of the flight frequency, this should result in a better accuracy due to a better representation of the reality in the dynamic graph. Thus, $J_t$ accounts for the frequency of flights on day *t* and is therefore dynamic, rather than static as in the previous cases.

### Measuring early warning signal performance

Unlike all the previous papers in the literature, which only make a visual interpretation, we implement a mathematical approach to the performance of the early warning signals regarding the daily confirmed cases. The methodology used to compare the different methods under consideration, can be divided into two parts:

1. First, we use the $R^2$ of a linear regression between the early warning signal shifted *x* days and the confirmed COVID-19 cases to determine the accuracy of the signal given an anticipation *x*. Thanks to the use of the $R^2$, we are sure that the value will always be in the range [0, 1] easily transformed into a percentage.
2. Second, we must identify how much the signal has to be shifted compared to the confirmed COVID-19 cases for the best possible fit. To do this, we run tests increasing the number of days the signal is shifted from 0 to 30 and we select the option that provides the best fit based on the $R^2$. As an alternative to the $R^2$, we will also use another measure of fit, the *Dynamic Time Warping* (DTW)^38,39^ distance between the two time series. Note that both signals are scaled to the range [0, 1], otherwise the resulting distances could be so great that they could lead to over-fitting problems. Thus, two possible shifts accounting for the $R^2$ and DTW, respectively, will be available.

Regarding the first step, like any simple linear regression, its objective is to approximate the parameters followed by $Y = \alpha + \beta X$ to explain the relation between the dependent or response variable *Y* and the independent variable *X*. The variable *Y* will be the daily confirmed COVID-19 cases, while the variable *X* will be the early warning signal to be checked. Simple linear regression makes the following assumptions about the parameter variables and their relationship: linearity (the mean of the response variable is combinatorially linear to the regression coefficients and the independent variable), homoscedasticity (constant variance without clear behavioral patterns), independence and normality of residuals.

It is important to note that these assumptions have been successfully checked for the problem under consideration for each early warning signal. Here we provide an example for only one early warning signal based on the number of edges with a complete adjacency and a threshold of $r=0.5$.

**Figure 1:**
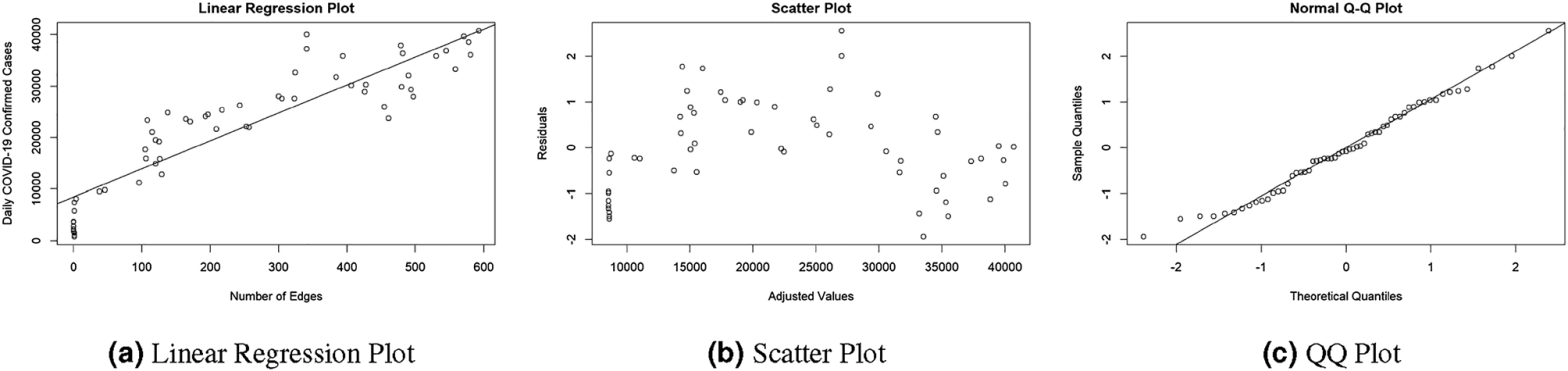
Illustrating the verification of the linear regression’s assumptions.

The corresponding coefficient of determination is $R^2=0.816$, which is relatively high. Figure 1 illustrates the assumptions of linear regression: linearity, homoscedasticity and normality. Regarding linearity, Fig. 1a plots all the points used to build up the regression model, i.e. the early warning signal shifted *x* days (in this case, *number of edges*) and the confirmed COVID-19 cases, with the resulting adjusted line. We find that a linear regression model fits the point cloud well, and therefore we can accept linearity. The residuals of the regression model are plotted in Fig. 1b, where homoscedasticity can be assumed, since no clear pattern can be detected in the the residual variability. Moreover, the Breusch-Pagan test returns a $p-value = 0.6754$ which is above 0.05, therefore not rejecting the null hypothesis (homoscedasticity).

Finally, the normality of residuals can be visually checked by means of the QQ plot shown in Fig. 1c, where quantiles of the residuals are plotted against the quantiles of a normal random variable. As all the points are mostly above the line, we assume the normality is met. Alternatively, the Kolmogorov-Smirnov test was performed, which returned a $p-value = 0.9487$, thus not rejecting the null hypothesis (normality).

### Results

First of all, it is important to note that the behavior of the network statistics and *DNM* methods are completely different. Most of the network statistics tend to mimic the trend of the confirmed COVID-19 somewhat in advance, whereas *DNM* methods only fluctuate or have huge peaks prior to the outbreak.

**Figure 2:**
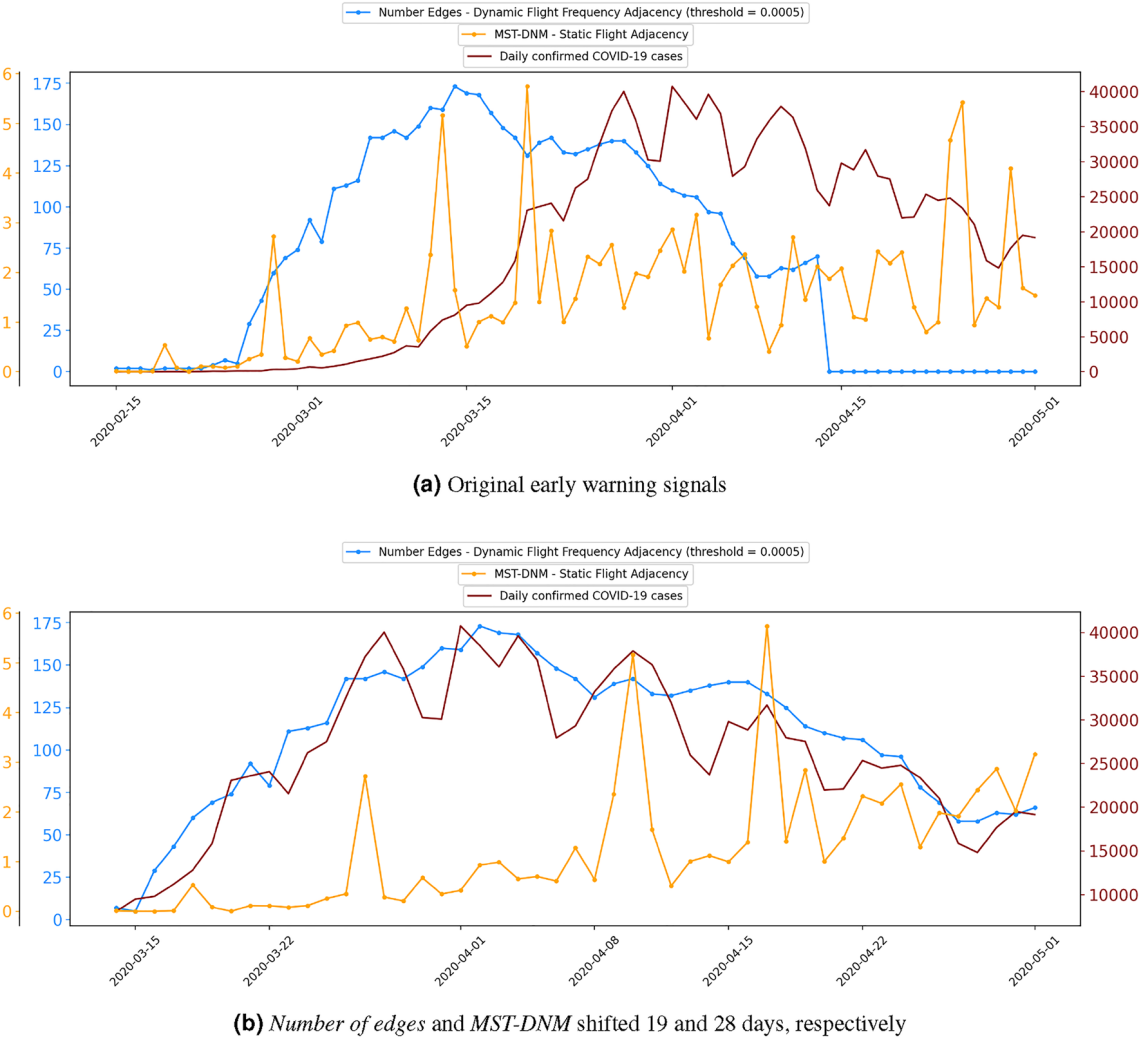
Comparing early warning signals by *number of edges* and *MST-DNM*.

This point is illustrated in Fig. 2a, where the orange early warning signal is generated with *MST-DNM* (explained in Section "Dynamic Network Marker (DNM)") using a *static flight adjacency*, the one in blue represents the *number of edges* method (defined in Equation (2)) with a *dynamic flight frequency adjacency* (using a threshold of 0.0005), and the number of daily confirmed cases in the whole of Europe is plotted in dark red. It shows how the *DNM* signal fluctuates a lot with many more peaks than the signal based on network statistics. However, if the early warning signal generated with *MST-DNM* (defined at the end of Section "Dynamic Network Marker (DNM)") is shifted forward 28 days and the signal based on the number of edges is advanced 19 days, see Fig. 2b, we find that there is a correlation between both early warning signals and the number of daily confirmed COVID-19 cases (obtained from WHO). As previously mentioned, the blue signal follows the trend of the confirmed COVID-19 cases, while the orange signal can somehow detect some of the peaks in the confirmed COVID-19 cases, even though it does not emulate the COVID-19 trend.

Let us analyse the behavior of the Dynamic Network Markers (*DNM*) in detail. First, it is important to note that, whereas the *MST-DNM* method only outputs a time series as an early warning signal, whose performance could be easily analysed and compared with network statistic-based methods or the daily confirmed COVID-19 cases, the Shortest Path (*SP-DNM*) and the Landscape (*L-DNM*) methods output multiple time series, which makes the interpretation of the results very difficult.

*MST-DNM* would appear to be the most promising *DNM*, and this is primarily because its output is a one-dimensional time series. As previously mentioned, this signal does not emulate the COVID-19 trend, instead it detects the peaks. However, it is not able to detect all the main peaks. This is a problem, since it is not possible to ascertain which peaks will be undetected. Additionally, the heights of the peaks do not match up with the increase in the number of infected people. As shown in Fig. 2b, the first peak of the orange signal, around the 27 March 2020, is half the height compared to the peak around the 17 April 2020. This should not be the case considering that the first peak represents around 40,000 confirmed new cases, whereas the second one accounts for around 30,000.

Unlike the *MST-DNM*, *SP-DNM*M outputs multiple time series, specifically between 1 and $(N(N - 1)) / 2$ time series, whose maximum matches up with the maximum number of edges of a complete undirected graph with N edges, although four time series should be enough if correctly selected^30^. Each time series determines the shortest path between two nodes, and the best option is usually to select the paths between the main cardinal points (northernmost-southernmost, easternmost-westernmost, northwesternmost-southeasternmost, southwesternmost-northeasternmost).

**Figure 3:**
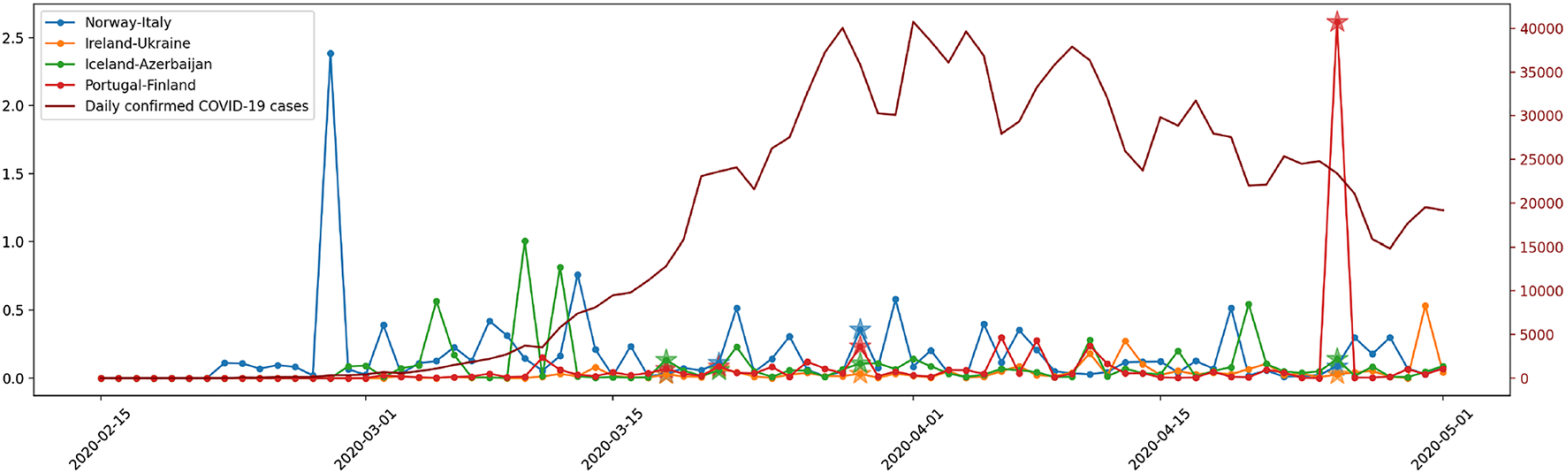
*SP-DNM* with *static flight adjacency*.

Figure 3 displays the *SP-DNM* early signals with the four mentioned cardinal connections, which in our study correspond to Norway-Italy, Ireland-Ukraine, Iceland-Azerbaijan, Portugal-Finland, respectively. Clearly, the inclusion of multiple time series greatly hinders the study. The stars in the time series point out the warning peaks using the fold change measure, highlighting a noticeable change between one measurement and the next. In this example, a star only appears if an increase of $10\%$ occurs in the four resulting time series at the same instant of time. There does not appear to be a very good match between the marked points and the real COVID-19 peaks, not to mention the variability between the actual signals.

**Figure 4:**
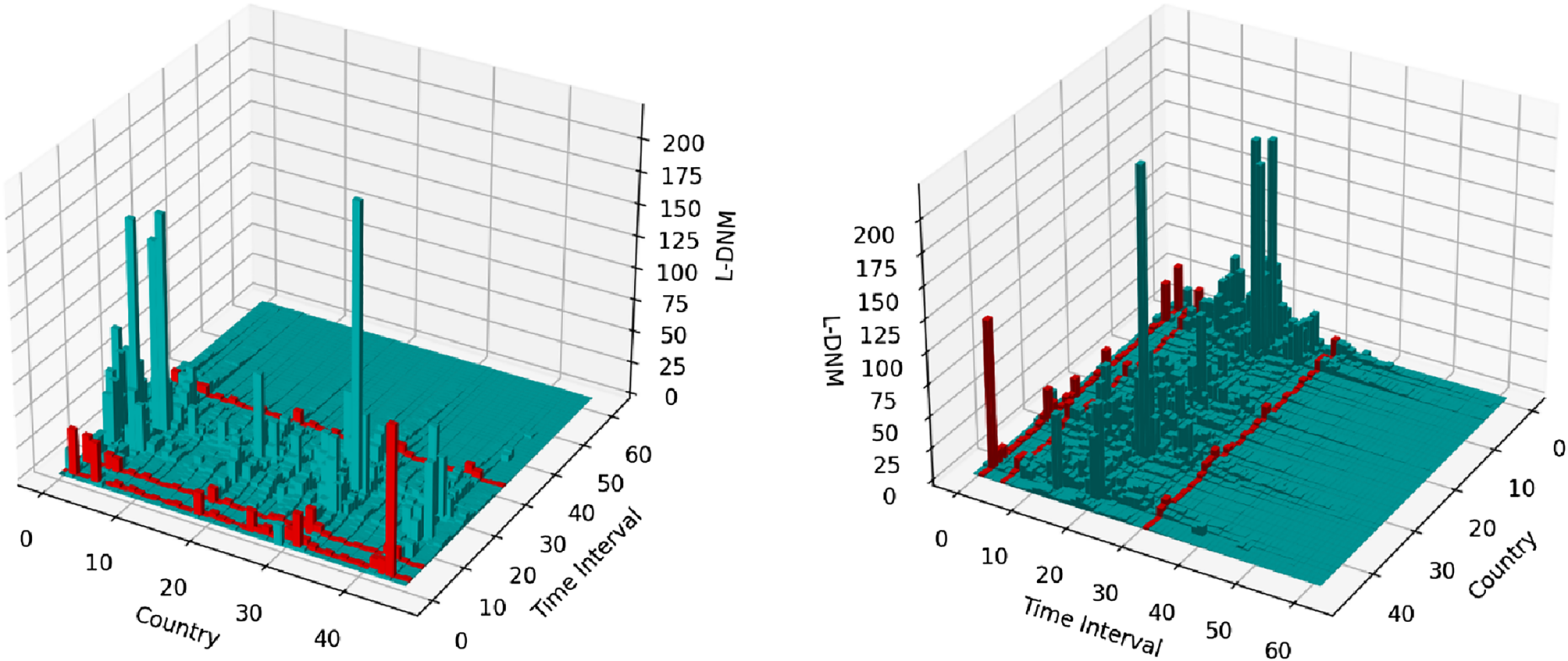
*L-DNM* with *static flight adjacency*.

Following the line of the previous *DNM*, *L-DNM* does not output a single time series, instead it outputs as many time series as the countries under consideration (in our case 46). Due to the large number of time series, a line plot does not properly represent the data. Therefore, a 3D bar plot (also known as landscape) must be used, as shown in Fig. 4 with *static flight adjacency*. The high dimensionality of the output makes its evaluation in relation to the confirmed cases of COVID-19 very difficult. On this ground, the only means of determining the early warning peaks is again with the fold change measure. The red line bars in Fig. 4 point out that all the time series increase at least a $100\%$ from one time instant to the next. Again, it is very difficult for a single value to determine how accurate this measure is.

As a consequence of these findings, and in light of the fact that all the *DNM* behave similarly, we have opted to focus exclusively on network statistics. This does not downplay the importance of *DNM* signals. However, it is more difficult to mathematically demonstrate their power.

Table 1 shows the performance of the network statistic-based methods (at the rows) described in Section "Network statistics" using the four types of adjacency matrices (at the columns) described in Section "Incorporation of air traffic data to the dynamic networks". Each method is assigned 2 rows, the first corresponding to the results when the best accuracy (*Best* $R^2$) is considered and the second one corresponding to the results accounting for the best shift (the more anticipation the better). In addition, each main column (adjacency matrix) is decomposed into 3 subcolumns, corresponding to shift values (best shifts when the $R^2$ and DTW are considered, respectively, separated by a slash) measured in days, $R^2$ values and the threshold (*r*) used in the application of the corresponding network statistic-based method.

The table cells have been shaded using four colors to facilitate the interpretation of the results: green is used when the result is excellent in terms of both accuracy and anticipation ($R^2\ge 0.85$ and the minimum of best shifts $\ge 18$); orange means that the result is still good, but the accuracy or the anticipation is slightly weaker ($R^2\in [0.8,0.85)$ or minimum *best shift* $\in [16,18)$); white covers mediocre results ($R^2\in [0.65,0.8)$ or minimum *best shift* $\in [13,16)$); and red indicates results that were directly discarded ($R^2<0.65$ or the minimum *best shift* <13).

Taking a quick look at the table and the shading of the cells, the first conclusion that we can reach is that there is no specific method that stands out over the others for any type of adjacency matrix in terms of $R^2$ and anticipation. Furthermore, if the cells shaded in red are discarded, most methods achieve their best accuracy with a low threshold (except the *clustering coefficient* with a *static complete adjacency* matrix), and the best anticipations are reached using high threshold values. If the $R^2$ increases, the shift generally decreases, and vice versa. On this ground, the tuning of the threshold must be tuned by a professional, who should take into consideration what is more important for their interests: accuracy or anticipation.

In addition, the *number of edges* and the *network density* methods perform equally for the first three static adjacency types both in terms of $R^2$ and anticipation. This means that the signals are proportional but not that they are equal. However, as expected, the results are slightly different when the *dynamic flight frequency adjacency* is used, given that, thanks to dynamic adjacency, the network density divides the number of edges between the number of nodes that vary over time.

If we analyze the specific values in the green cells, the first thing we notice is that the anticipations vary within the interval [18, 21] days, while the $R^2$ is within the interval [0.875, 0.920]. This implies that they all provide very good results and that no method outperforms the rest, since method with the highest $R^2$ (*number of edges* method with *dynamic flight frequency*) is outperformed in terms of anticipation by some of the others, and the methods with the highest anticipation (21;19 days) are slightly worse in terms of $R^2$.

Looking at the four types of **adjacency information** (four main columns in the table), we find that the results output for the *dynamic flight frequencies* (last main column) appear to be better than those for the other types of adjacency. The corresponding values are shaded in green (good values) for the four first methods (we do not account for the last two methods shaded in red for the 4 the four types of adjacency information) when the best $R^2$ is considered. Moreover, good results are also achieved for the *number of edges* method when the best anticipation is considered. However, goods results are output only for the *number of edges* and *network density* methods, when the *static flight adjacency* and the *static flight frequency* are considered to account for the best $R^2$ (although accuracy and anticipation values are slightly outperformed by *dynamic flight frequencies*), whereas only the *clustering coefficient* outputs good results when both the *static complete adjacency* (in addition to *static flight adjacency*) are taken into account for the best $R^2$.

We can conclude that using flight frequency data to compute the adjacency matrix increases the method performance in terms of both accuracy ($R^2$) and anticipation. Furthermore, it is remarkable how the use of a dynamic rather than static adjacency leads to a noticeable improvement. It becomes clear that the introduction of air traffic data to the methods achieves a more faithful representation of reality, which they can then exploit. Note, however, that the processing of air traffic data requires a more complex implementation and greater computational time, especially in the case of dynamic adjacency.

If no flight data is available, it is still possible to achieve good results with *static complete adjacency* and *static flight adjacency*. The only problem is that threshold tuning is much trickier, because its increase might reduce the accuracy and increase the anticipation and its decrease might do the opposite. The *clustering coefficient* is less affected, which explains why it can achieve slightly better results without the use of other more complex adjacency types.

Another noteworthy fact is that 10 out of the 11 cells shaded in green correspond to analyses considering the best $R^2$.

Moving on the **methods under consideration** (rows), we find that the *Forman-Ricci curvature* and the *assortativity coefficient* are outperformed (shaded in red in all but one of the cells) by the rest of the methods for any type of adjacency matrix. Therefore, they can be discarded for further analysis. The explanation is that these methods are not always able to generate a numerical number for each instant of study, preventing the generation of early warning signals. This leads to time series with Not A Number (*NaN*) values in some instances, which must be replaced before analysing linear regression. The range of possible values for the *assortativity coefficient* is $[-1, 1]$, but the limits are very rarely achieved. On this ground, we have replaced *NaN* values by the minimum value in the time series. On the other hand, the range of possible values in the Forman-Ricci curvature is not close, namely $[-x, x] \forall x \in \mathbb {N}$. Therefore, all *NaN* values have been replaced by 0. These replacements make the signals more unstable, which can be seen perfectly in the resulting shifts in the table, regardless of the type of adjacency, where the values are either very low and close to 0, or very high and close to the maximum of 30 days. Another problem with the *Forman-Ricci curvature* is that there are usually many ups and downs in the signal at the beginning, thus lacking progress in growth.

The four remaining methods output good results when the *dynamic flight frequency*y is taken into account for the best $R^2$. If we analyze that table column in detail, we find that the *PRS* method and the *clustering coefficient* are very slightly outperformed by the other two methods in terms of accuracy, with very similar values for anticipation. Moreover, the *PRS* method only outputs good results when the dynamic flight frequency is considered. Thus, we could conclude that the *PRS* method is outperformed by the other three methods.

Furthermore, the *number of edges* and the *network density* output good results when both the *static flight adjacency* and *static flight frequency* are considered. However, these outputs are slightly outperformed by the two methods with *dynamic flight frequencies*. The same applies for *clustering coefficient*, shaded in green, with *static flight adjacency* accounting for the best $R^2$, which is, however, slightly outperformed by the results output when *dynamic flight frequencies* are considered.

*Clustering coefficient* is the only method with good results when the *static complete adjacency* is considered, accounting for the best $R^2$. These results are again slightly outperformed by *dynamic flight frequencies*.

Finally, if we try to identify the best combination method-adjacency in the table (best cell), there is no combination that clearly outperforms the others. All combinations shaded in green output good results, with only minor differences from one to the other..

However, if we prioritize anticipation over accuracy ($R^2$), the *number of edges* and the *network density* methods with *dynamic flight frequency* could be selected (with $R^2 = 0.920$ and 0.915, and shifts 19;18 and 20;20, respectively)). The *number of edges* (shifts 21;19) or *network density methods* (shifts 21;19), both with *dynamic flight frequency* and very similar $R^2$ values, could be selected when the accuracy is prioritized. Based on the above, we could conclude that the *number of edges* and the *network density* methods with *dynamic flight frequency* should be used.

**Table 1:**
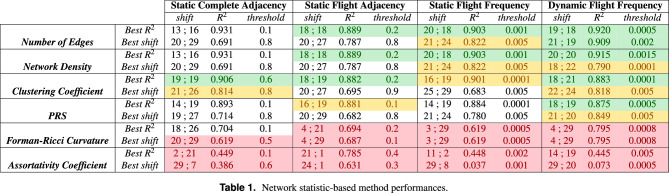
Network statistic-based method performances.

**Figure 5:**
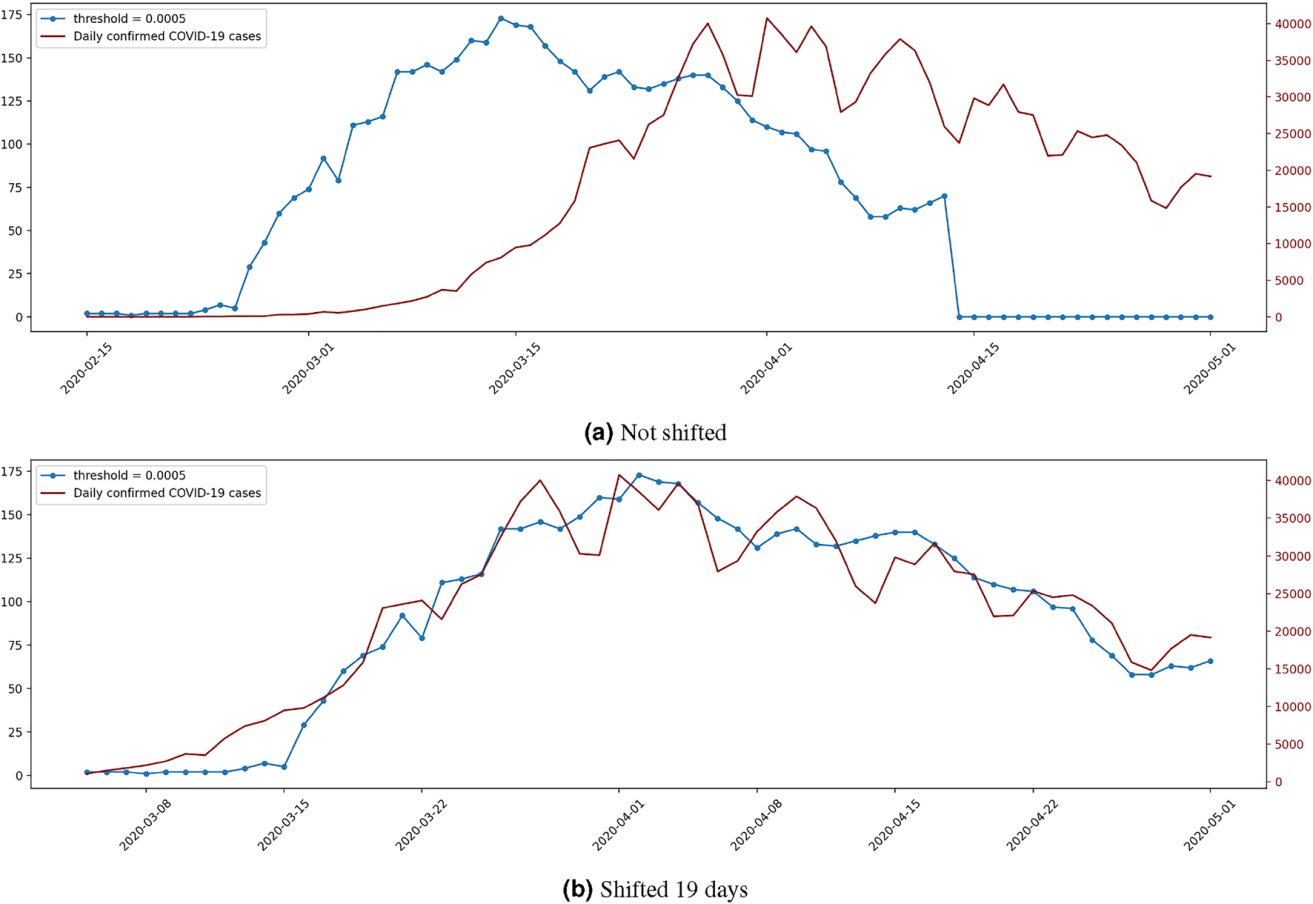
Number of edges - dynamic flight frequency adjacency.

Figure 5 plots in blue the early warning signal output by the *number of edges* method with *dynamic flight frequency* and a threshold of 0.0005 against the confirmed daily COVID-19 cases ($R^2=0.920$, shift 19 days). The left *Y* axis represents the number of edges corresponding to the early warning signal, whereas the right *Y* axis represents the number of confirmed daily COVID-19 cases in Europe. The signal is not shifted at all. Accordingly, each instant *t* has the prediction trend for the day $t+19$.

Note that the early warning signal generated output value 0 from 14 April 2020 since all flights in Europe were banned 19 days later, which is what leads to an adjacency matrix with 0s in all its elements. This is not a problem since the main objective is the early detection of a pandemic rather than a long-term prediction. However, simple changes can be applied if a long-term prediction is desired, for instance, by changing the adjacency type when no flights are detected.

Figure 5b plots, again in blue, the same early warning signal as in Fig. 5 but shifted forward 19 days. The overlap between the generated signal and the COVID-19 cases is clear, and while they are on different scales, most peaks are detected.

## Conclusions

In this paper, different early warning signals for detecting COVID-19 outbreaks in Europe have been reviewed and compared. Two classes of methods have been identified: *network analysis*-based methods, in which dynamic networks are generated and analyzed on the basis of different network global properties or well-defined formulas; and *dynamic network markers* (*DNM*), which are model-free methods derived from dynamic network biomarkers used to detect the tipping point in some diseases, which do not require any training and work well even with few samples.

Air traffic information has been incorporated into the analysis by means of four alternative adjacency matrices including static complete, static flight, static flight frequency and dynamic flight frequency adjacency information.

First, *DNMs* were discarded for further analysis. Their behavior is completely different from *network analysis*-based methods, which tend to mimic the trend of the confirmed COVID-19 cases somewhat in advance. *DNM* methods only fluctuate or have huge peaks prior to the outbreak. Although the *MST-DNM* method only outputs one time series as an early warning signal, *SP-DNM* and *L-DNM* output multiple time series, which makes it very difficult to interpret their results. Moreover, *DNMs* are not able to detect all the main peaks and the height of the peaks if the early signals do not match up with the increase in the number of infected people.

Regarding the four types of adjacency information considered to analyze the *network analysis* based methods, we can conclude that using flight frequency data to compute the adjacency matrix increases the method performances, and so even more if dynamic adjacency is considered, with the drawback of a higher computational time expenditure. This makes sense since the introduction of the air traffic data to the methods derives a more faithful representation of reality, which can be exploited by the methods.

Regarding *network analysis*-based methods, a first conclusion is that the *Forman-Ricci curvature* and the *assortativity coefficient* are outperformed by the rest of the methods for any type of adjacency matrix. They can thus be discarded.

In addition, no specific method clearly stands out over the others for any type of adjacency matrix in terms of accuracy and anticipation. There are only minor differences between the results of methods identified as good. However, the *number of edges* and the *network density* methods with *dynamic flight frequency* output the best results when both accuracy and anticipation are prioritized.

We propose as a future research line the incorporation of information about each country’s testing capacity to the corresponding number of confirmed cases to better reflect the pandemic evolution.

## Data Availability

Information about confirmed COVID-19 cases has been obtained from the two most reliable sources, the *Johns Hopkins University* (JHU)^35^ and the *World Health Organization* (WHO)^34^, which are accessible free of charge. The datasets generated and/or analyzed during the current study are not publicly available since they were extracted from a private graph database (Neo4j) provided by the Spanish Research Center CRIDA (*Centro de Referencia de I+D+i ATM*), but are available from the corresponding author on reasonable request.

## Code availability

Both data and code are open access in a Python library available at: https://github.com/aanxel/EWarningSignals^40^.
